# Creep and Creep Recovery Response of Load Cells Tested According to U.S. and International Evaluation Procedures

**DOI:** 10.6028/jres.102.024

**Published:** 1997

**Authors:** Thomas W. Bartel, Simone L. Yaniv

**Affiliations:** National Institute of Standards and Technology, Gaithersburg, MD 20899-0001

**Keywords:** creep, creep recovery, force measurement, load cell

## Abstract

The 60 min creep data from National Type Evaluation Procedure (NTEP) tests performed at the National Institute of Standards and Technology (NIST) on 65 load cells have been analyzed in order to compare their creep and creep recovery responses, and to compare the 60 min creep with creep over shorter time periods. To facilitate this comparison the data were fitted to a multiple-term exponential equation, which adequately describes the creep and creep recovery responses of load cells. The use of such a curve fit reduces the effect of the random error in the indicator readings on the calculated values of the load cell creep. Examination of the fitted curves show that the creep recovery responses, after inversion by a change in sign, are generally similar in shape to the creep response, but smaller in magnitude. The average ratio of the absolute value of the maximum creep recovery to the maximum creep is 0.86; however, no reliable correlation between creep and creep recovery can be drawn from the data. The fitted curves were also used to compare the 60 min creep of the NTEP analysis with the 30 min creep and other parameters calculated according to the Organization Internationale de Métrologie Légale (OIML) R 60 analysis. The average ratio of the 30 min creep value to the 60 min value is 0.84. The OIML class C creep tolerance is less than 0.5 of the NTEP tolerance for classes III and III L.

## 1. Introduction

For the past 5 years the Force Group of the Automated Production Technology Division of the National Institute of Standards and Technology has been performing load cell testing according to the National Type Evaluation Program (NTEP) as specified in the National Conference of Weights and Measures Publication 14 [1]. These tests, which at NIST are performed using primary force standards, determine certain metrological characteristics of load cells submitted by load cell manufacturers desiring to certify their load cell families as compliant with accuracy class requirements specified in NIST Handbook 44 [2].

The NTEP testing is performed with the load cells enclosed within environmental chambers designed to control the temperature of a load cell, its cable, and mounting fixture while calibrated forces are applied by deadweights. The metrological characteristics that are determined by NTEP testing include load cell linearity, hysteresis, repeatability, temperature effect on minimum dead load output, and creep, all evaluated over a temperature range of −10 °C to 40 °C.

This paper summarizes the results of analyses of the creep responses obtained from load cells tested at NIST. As described in detail in the next section, load cell creep is the difference between an initial response after a force change and the response at a later time. The purposes of the analyses presented herein were: (1) to determine how the creep response (which follows the sudden application of force) compares to the creep recovery response (which follows the sudden release of force); and (2) to compare the creep results from the NTEP procedure with those from the corresponding international standard, the Organization Internationale de Métrologie Légale (OIML) R 60 [3]. The NTEP and OIML R 60 specifications, while similar, have significant differences.

## 2. Load Cell Creep Response

When the applied force acting upon a force transducer, such as a load cell, is changed rapidly to a new level and then remains constant, the force indicating system of the transducer yields a value that drifts, or creeps, with time before reaching equilibrium (providing that the transducer is sufficiently well-behaved to reach a stable value). As described By Pontius and Mitchell [4], this creep is largely attributable to thermoelastic effects: the adiabatic heating and cooling of elastic load supporting elements within a load cell as they undergo deflection in response to changes in the applied force. A rheological model for load cell behavior by Mitchell and Baker [5] shows that the load cell output following a sudden application (or release) of force can be described as a function of time by

$$r=a_{0}+\sum a_{i}e^{-b_{i}t},$$ (1)

where *r* is the load cell output, or response, *t* is time since the force application, and *a*_0_ is the equilibrium response as *t* becomes very large. The number of significant terms depends on the number of significantly different contributors of thermoelastic effects; the values of the coefficients *a_i_* and the time constants *b_i_* depend on the complex interactions of the load supporting elements (their associated local adiabatic sources/sinks and heat flow parameters), the strain gauges and adhesives, and the thermal compensation and other elements in the electrical network. In addition, the values depend upon the loading history of the transducer, such as the period of time since the previous incremental change in applied force, and the magnitude and direction of this change. The coefficients may be of either sign; the time constants are always positive.

For most force measurement applications, the user of a load cell assumes a one-to-one correspondence between the applied force and the load cell indicator reading. Thus the time variation in the response due to creep, represented by the summation in Eq. (1), is a source of error in the determination of the applied force. A correction for creep is possible if the creep characteristics of the load cell are known and the time between the application of force and the reading of the indicator is controlled. Typically, the magnitude of the creep is a few hundredths percent of the applied load; the time before equilibrium may vary among load cells from minutes to hours.

For commercial weighing applications in the United States and many other countries, creep is controlled through tolerance limits that load cell manufacturers must meet for certification. The National Conference of Weights and Measures limits the 60 min creep for load cells being tested for NTEP certification to a tolerance that is equivalent to about 0.03 % to 0.05 % of the applied load (90 % to 100 % of cell capacity), depending upon classification parameters. The Organization Internationale de Métrologie Légale limits the 30 min creep for OIML R 60 class C (the class which most closely corresponds to the current NTEP classes) to about 0.007 % to 0.035 % of the applied load (also 90 % to 100 % of capacity). In addition, OIML R 60 limits the allowable creep that occurs from *t* = 20 min to *t* = 30 min to about 1/5 the value of the 30 min creep tolerance. The NTEP certification procedure accepts a return-to-zero creep test, denoted as creep recovery in this paper, in lieu of a creep test if test equipment limitations make the creep test impractical. The OIML procedure does not accept such a substitution; however, it requires a measurement of the minimum load output return (MLOR), which is the change in the minimum load reading before and after the 30 min application of a capacity load. The OIML class C tolerance for MLOR ranges from 0.005 % to 0.025 % of the applied load (90 % to 100 % of capacity).

It is the comparison of creep and creep recovery, as well as the 60 min creep with creep over shorter time periods, that this paper addresses. It is beyond the scope of this work to predict the actual values of the equilibrium response a_0_, the transient coefficients *a_i_*, or the time constants *b_i_* in Eq. (1) for any particular load cell from its structural dimensions, the mechanical, elastic and thermal properties of its components, and the characteristics and assembly of the elements of the strain gauge bridge network.

## 3. NTEP Creep Test Procedure

All creep test data used for the comparisons described above were performed at NIST according to the procedure given in the NTEP specification [1]. Three of the six NIST deadweight machines were used to perform the tests described herein. These three machines have capacities of 498 kN (112 klbf), 113 kN (25.3 klbf), and 2.2 kN (500 lbf). These machines, having weights and loading frames made of stainless steel, are described in detail in Refs. [6,7]; therefore, only a short description of the machines is given below. The combined standard uncertainty (estimated standard deviation) in the applied forces due to uncertainties in the adjustment of the weights and variations in air density is 0.0005 % of the nominal applied force.

**Fig. 1:**
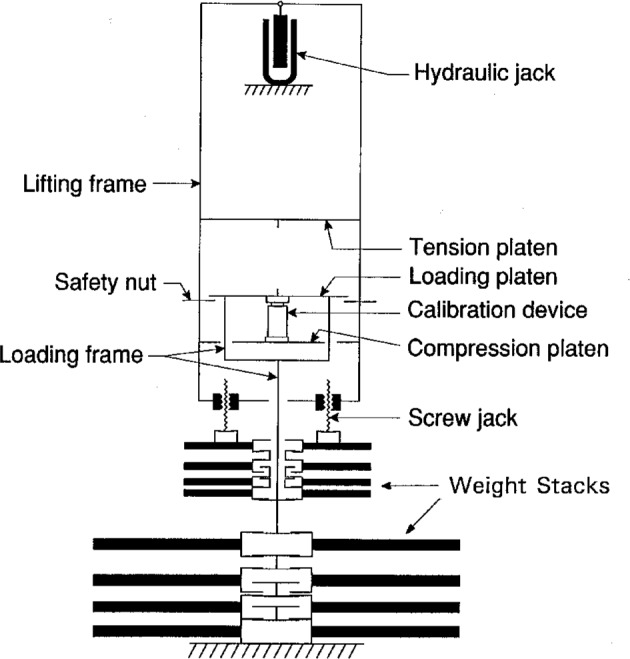
Schematic diagram of the NIST 498 kN (112 klbf) deadweight machine.

The 498 kN deadweight machine, shown schematically in Fig. 1, utilizes two weight stacks: a large stack consisting of ten weights, each of which are adjusted to produce a force of 44.48 kN, and a small stack of nine weights each adjusted to produce a 4.448 kN force. The loading frame, which constitutes the machine’s minimum load, produces a calibrated force of 13.34 kN. The large weights are applied sequentially by raising the lifting frame with the hydraulic jack, thus raising the loading frame as the lifting force acts through the force transducer. The small weight stack is operated independently of the large weight stack, with the small weights applied by screw jacks which lower them sequentially onto the loading frame. The total applied force is thus due to the sum of the weight of the loading frame and the weights from the two stacks being borne by the loading frame.

The 498 kN machine has been fitted with an auxiliary hydraulic jack to accomplish the transfer of the loading frame, loaded with deadweights equivalent to the load cell capacity, onto the loading point of the cell within one second or less. This enables the loading time requirements of the NTEP creep test to be met, overcoming the time limitation otherwise imposed by the sequential weight-lifting mechanism of this machine. On the two smaller machines, built-in mechanisms to raise or lower the weight frame serve this same purpose.

**Fig. 2:**
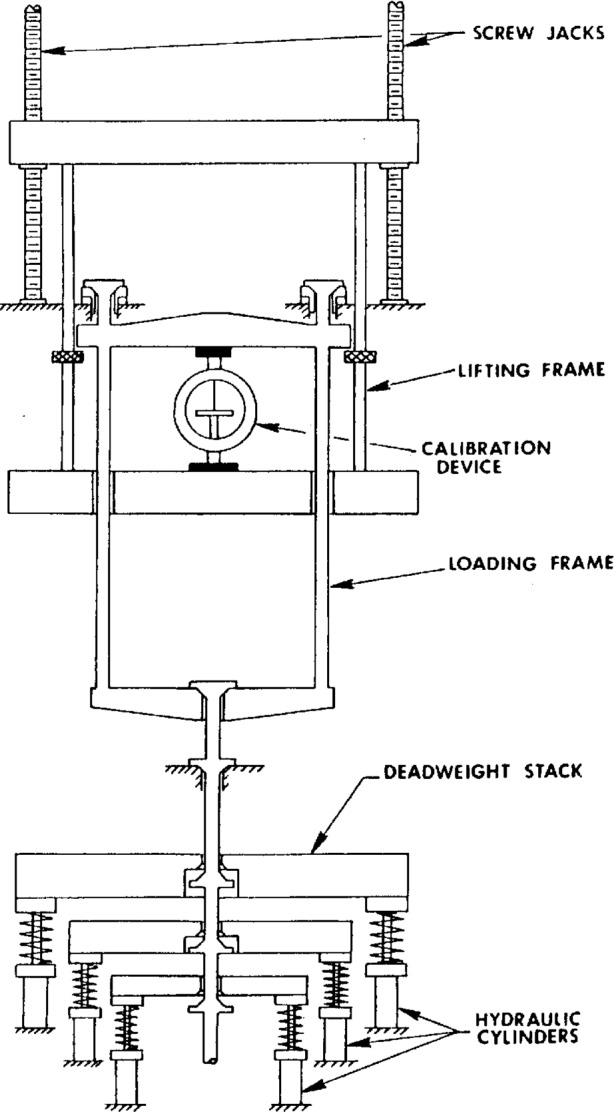
Schematic diagram of the NIST 113 kN (25 klbf) deadweight machine.

The 113 kN deadweight machine, shown schematically in Fig. 2, utilizes eleven weights in graduated increments adjusted to produce forces from 444.8 N to 22.24 kN. The loading frame generates a minimum force of 1779 N. Each weight can be applied to the loading frame independently of the other weights by hydraulic cylinders which compress the springs which otherwise support the weights in an unloaded position. A pneumatically operated stabilizing mechanism has been installed to enable these weights to be changed without excessive swinging; thus the original operational limitation of return-to-zero loading, as described in Ref. [7], has been overcome, permitting the monotonically ascending and descending force sequences required by NTEP.

The 2.2 kN deadweight machine is schematically similar to the 113 kN machine, and has eight weights in graduated increments to produce forces from 22.24 N to 889.6 N. The loading frame generates a force of 44.48 N. The weights are applied independently of each other to the loading frame; the actuation, originally by manual operation, is now accomplished by means of pneumatic cylinders. A stabilizing mechanism has been installed which is similar to that of the 113 kN machine.

Environmental chambers for the three machines listed above have been specifically constructed for NTEP testing, providing for thermal isolation of a load cell and any associated fixture from the machine frame while calibrated forces, generated by the deadweights, are applied. For the two smaller machines, separate chambers are available to provide for either compression or tension loading. Since large capacity tension devices are rarely submitted for NTEP certification, the chamber for the larger machine has been designed for compression loading only. Heating and cooling is done through computer-controlled bath units, and several sensors allow for digital input of air temperature, surface temperatures of the load cell body and loading blocks, and the barometric pressure. All electrical control functions for the deadweight machines are interfaced to allow computer control of the weight applications (while still maintaining the original manual control capability). The load cell output is sampled by an 8 1/2-digit digital multimeter operating in voltage-ratio measurement mode. The instrumentation and algorithms for implementing automated control of the deadweight machines, voltage-ratio indicators, and environmental chambers have been described by Yee [8]. These automated systems make it possible to perform the NTEP tests, each involving several temperature changes, completely under computer control.

The load cells included in this study are tested at each of three temperatures: 20 °C, −10 °C, and 40 °C. At each temperature, after thermal equilibrium is obtained, the creep testing is done as follows:

- The load cell, which has remained unloaded during the temperature transition and stabilization period, is exercised three times by the application of a load of 90 % to 100 % of capacity and returned to an unloaded condition; the load cell then remains unloaded for 1 h; an initial reading of the load cell indicator is taken with the load cell unloaded.
- The 60 min creep response is then obtained: a capacity load is transferred to the load cell and remains applied; twenty seconds later a reference reading of the load cell indicator is taken, with subsequent readings taken at one minute intervals for 60 min (two additional readings are taken during the first minute).
- At NIST, the creep recovery, or return-to-zero, response is then obtained, by unloading the load cell and taking readings in the same time sequence as was done for the creep response. (Creep recovery is not required by NTEP.)

For NTEP evaluation, the maximum 60 min creep, expressed here as a fraction of the maximum indicator reading, is calculated from

$$C=(r_{\max}-r_{\text{ref}})/(r_{\text{ref}}-r_{0}),$$ (2)

where *r*_0_ is the initial unloaded reading, *r*_ref_ is the 20 s reference reading, and *r*_max_ is the reading that gives the maximum value of |*r_n_* − *r*_ref_|; *r_n_* is the *n*th reading following the reference reading.

The maximum creep recovery value is calculated in a similar manner. The NTEP specification permits creep recovery data to be used for evaluation in lieu of creep data only when the creep response cannot be measured because of equipment limitations. This was occasionally the case at NIST when the movement afforded by the load cell mounting fixture made it impossible to maintain proper positioning of the deadweight machine weight frame after unloading and reapplying the creep test load. This problem has been eliminated through redesign of the auxiliary hydraulic lift that was installed on the 498 kN machine to implement the creep loading. Some manufacturer’s testing facilities, used for load cell development and production quality control, apply forces that are generated by hydraulic pressure rather than by deadweights; a creep recovery test must be used since such a system cannot maintain a sufficiently uniform force to test for creep over a 60 min time period.

The creep and creep recovery data obtained at NIST by the procedure described above make it possible to directly compare the load cell creep and creep recovery behavior.

## 4. Creep Response Curve-Fitting Procedure

In order to facilitate comparisons such as between creep and creep recovery and between 60 min and 30 min creep values, a nonlinear model, having the form of Eq. (1), was fitted by a least-squares method to the data for each response. In addition to yielding each response in a form that can be readily evaluated, this approach minimizes the effect of the random error inherent in the indicator readings, as discussed later in Sec. 5.2. In the earlier work that originally presented the creep response model [5], such curve-fitting was done by means of an optimization search algorithm [9] run on a mainframe computer. In the present work, a data analysis program developed at NIST [10-12] was used to perform the curve-fitting.

**Fig. 3:**
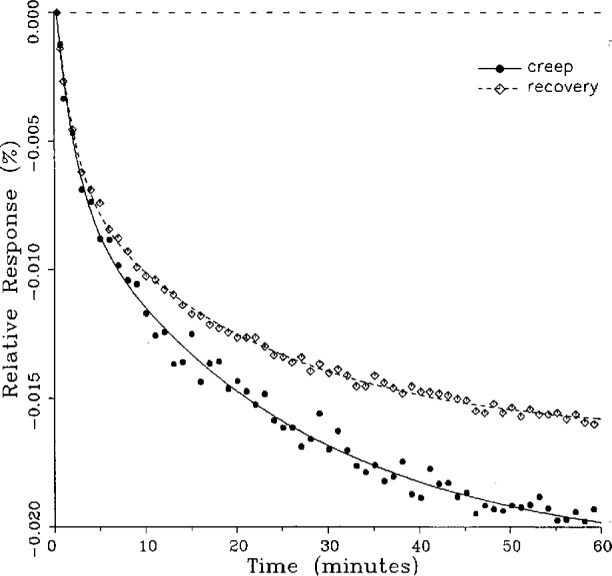
Creep response and creep recovery response of a shear-beam load cell of capacity 17.8 kN; temperature: 19.6 °C.

Plots of typical creep and creep recovery data for the same load cell for a single temperature are shown in Fig. 3 together with the fitted curves. The time *t* = 0 on the horizontal axis represents the instant that the creep test load is applied (for the creep response) or released (for the creep recovery response). The creep recovery data are displaced 60 min earlier in time in order to superimpose the curves. The first data point shown in the figure represents the 20 s reference readings for both the creep and creep recovery responses, plotted together on the baseline. The ordinates of the following points give the drift, or change, in the indicator readings relative to the reference point expressed as a percentage of the full load reading; i.e.,

$$Y_{n}=100(r_{n}-r_{\text{ref}})/(r_{\text{ref}}-r_{0}),$$ (3)

where *Y_n_* is the ordinate of the nth data point following the reference point in the creep response shown in Fig. 3, and *r_n_*, *r*_ref_, and r_0_ are the same as defined following Eq. (2).

The ordinates for the creep recovery response are calculated similarly, but have an additional factor of −1 incorporated into Eq. (3), thus allowing the creep and creep recovery to be directly compared in the same graph quadrant. Thus, a negative slope for the creep response, as seen in Fig. 3, represents a load cell reading that is decreasing with time (i.e., indicating a lessening load), while the same negative slope for the creep recovery response represents a load cell reading that is increasing with time.

The smooth solid curve shown in the figure is the fitted curve for the creep response relative to the 20 s reference point reading; it is given by

$$y=100(r-r_{\text{ref}})/(r_{\text{ref}}-r_{0}),$$ (4)

where *r* is the least-squares fit of Eq. (1) to the measured creep readings, limited here to two exponential terms. Thus *r* is given as a function of time *t* as

$$r=a_{0}+a_{1}e^{-b_{1}t}+a_{2}e^{-b_{2}t}.$$ (5)

The fitted curve for the creep recovery response, shown with the broken line in the figure, incorporates a factor of −1. The fitted curves are plotted here over a time period from 20 s to 60 min. Most of the curve-fitting calculations are limited to two exponential terms; a few, as discussed in the next section, incorporate three exponential terms.

## 5. Results

### 5.1 Demonstration of Fitted Curves

**Fig. 4:**
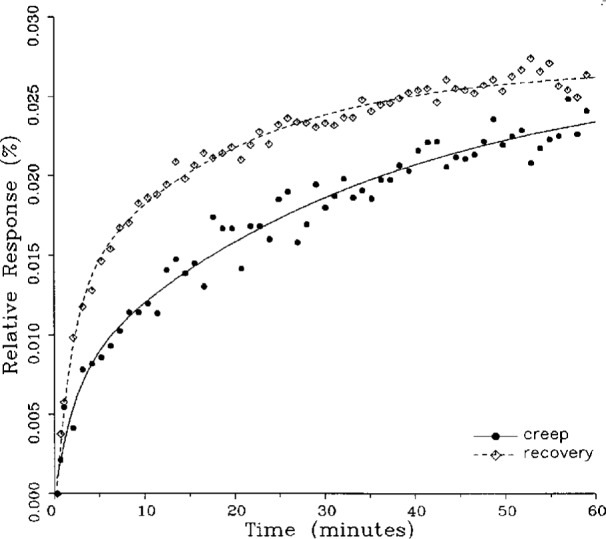
Creep response and creep recovery response of a single-point load cell of capacity 490 N; temperature: −8.9 °C.

**Fig. 5:**
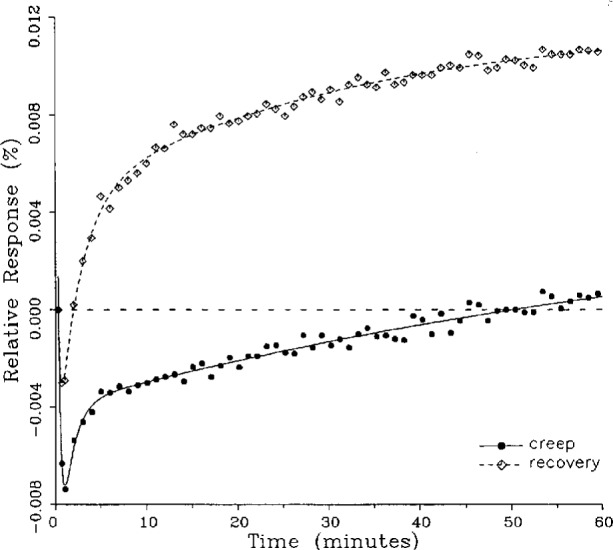
Creep response and creep recovery response of a canister load cell of capacity 445 kN; temperature: 19.8 °C.

**Fig. 6:**
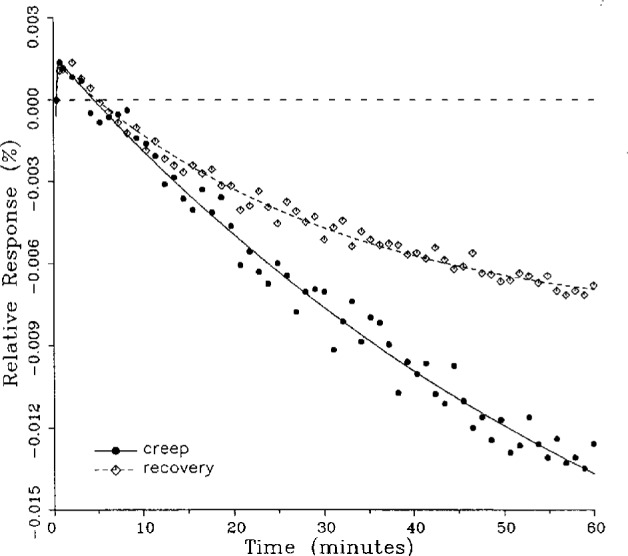
Creep response and creep recovery response of a shear-beam load cell of capacity 1.47 kN; temperature: 40.1 °C.

A number of creep and creep recovery responses, each superimposed with a plot of the equation generated by the least-squares fit, are shown in Figs. 4 to 9. The plots shown were selected to illustrate the great variation in the shapes of the responses realized among load cells. The degree to which the computed curves describe the actual data indicates that Eq. (1) is an adequate representation of load cell creep response. These figures represent a small portion of the 195 creep-creep recovery response pairs that have been fitted to Eq. (1); agreement for the cases that are not shown is, in general, as good or better than that shown in Figs. 3 to 9.

The fitted curves shown in Fig. 5 are calculated from three exponential terms in the summation in Eq. (1). This curve shows a very rapid initial transient; for such cases three terms are usually necessary to adequately describe the curve. The curves for the other figures are all generated from fits with two exponential terms. If the initial transient has a time constant of more than one minute, it was found that specifying a three-term fit results in a curve that essentially retraces the curve for a two-term fit.

The adequacy of the model defined by Eq. (1) in characterizing the creep response was judged primarily by visually comparing the data to the fitted curves. For those cases in which the random “scatter” of the points about the curve is low, the deviations are too small to be of relevance to the comparisons between creep and creep recovery being made in this study. For those cases in which the random variation is large, a plot of the residuals with time does not show any structure that would indicate that a mathematical model different from Eq. (1) should apply to these cases. The early portion of the curves for load cells having rapid initial creep transients could be better fit if more readings had been taken in that region.

No clear correlation between the load cell characteristics (type of construction, compression or tension loading mode, or cell capacity) and the shape of the creep response (direction of creep, fast or slow time constants, initial transient) is apparent from the measurements included in this study. In fact, significantly different creep responses are often seen in the same load cell at different temperatures. Figures 7 to 9 show such variations in the creep response with temperature for each of three load cells. The creep response is thus seen to be too complex to be generalized with simple rules of thumb. Analytical modeling tools, such as finite-element analysis, may be of value in predicting the creep response from a particular load cell’s design parameters.

### 5.2 Random Error Reduction

The use of the curve-fitting procedure enables a more accurate determination of the magnitude of the load cell creep by reducing the contribution of the random error in the indicator readings. For a device being evaluated on whether its creep response meets or exceeds specified tolerance limits, this serves to ensure that it is the actual device characteristic, rather than the random error in the measuring technique, that is being evaluated.

**Fig. 10:**
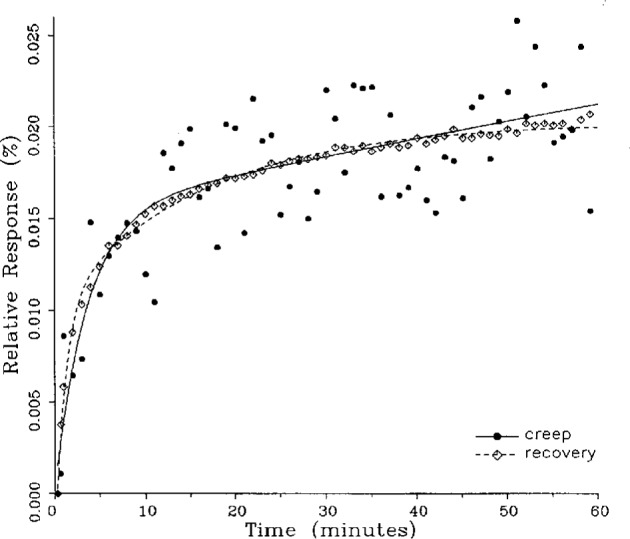
Creep response and creep recovery response of a shear-beam load cell of capacity 20 kN; temperature: 19.6 °C.

**Fig. 11:**
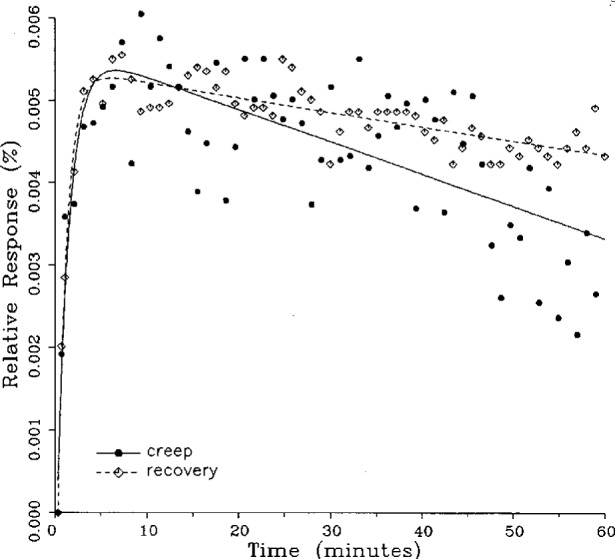
Creep response and creep recovery response of a shear-beam load cell of capacity 1.47 kN; temperature: 40.1 °C.

**Fig. 12:**
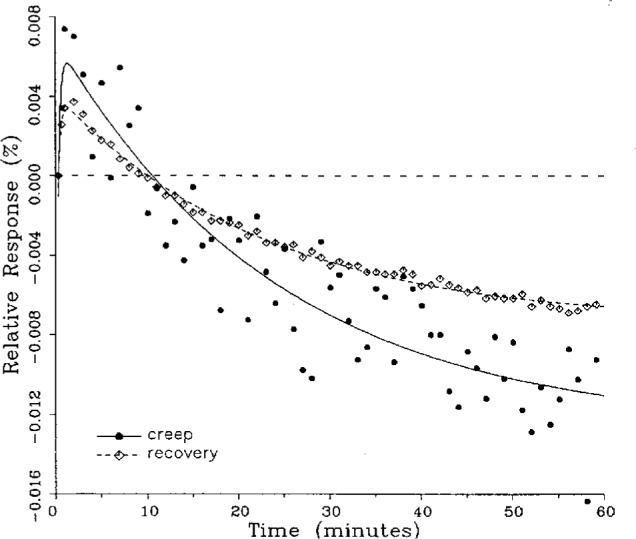
Creep response and creep recovery response of a shear-beam load cell of capacity 20 kN; temperature: 39.8 °C.

**Fig. 13:**
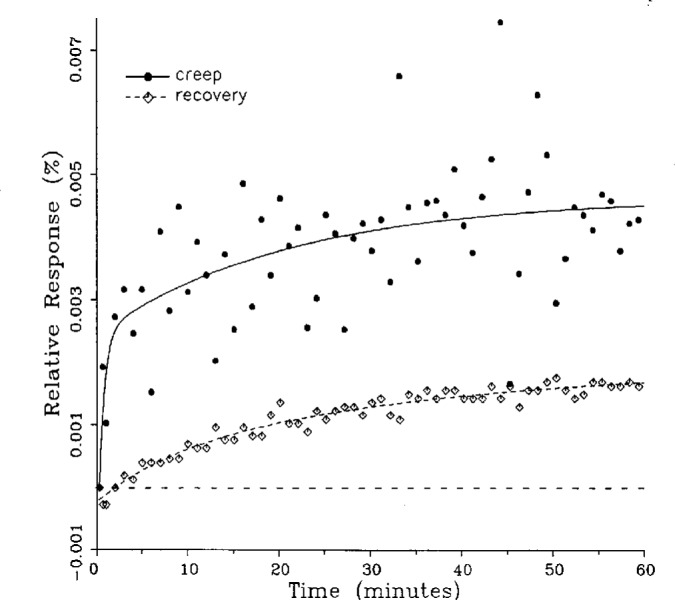
Creep response and creep recovery response of a C-shaped tension load cell of capacity 22.2 kN; temperature: −10.9 °C.

As can be seen from Eq. (2), the creep value *C* used for NTEP evaluation is computed from two data points: the reference point at *t* = 20 s, giving *r*_ref_, and the point of maximum creep, giving *r*_max_; thus, *C* will be uncertain by the combined uncertainty of both points. Figures 10 to 13 illustrate how this evaluation method can result in an unrealistically elevated value of *C*. For example, the single high reading at the 47 min point in Fig. 13 defines an NTEP creep value that more properly reflects a large random variation in the readings rather than an actual creep phenomenon. If the actual creep response is considered to be given by Eq. (1), in effect making use of all of the indicator readings *r_n_*, the corresponding creep value *C*′ is given by

$$C^{\prime }=[r(t_{\max})-r(t_{\text{ref}})]/(r_{\text{ref}}-r_{0}),$$ (6)

where *t*_ref_ = 0.33 min (20 s) and *t*_max_ is the value of *t* that gives the maximum value of |*r*(*t*) − *r*(*t*_ref_)| over the time interval from *t*_ref_ to 60 min. The denominator, being much larger than the numerator, is not significantly affected by the random variations and is thus left in the same form as in Eq. (2).

The ratio of *C*′/*C* for the four creep responses in Figs. 10 to 13 is 0.76, 0.81, 0.67, and 0.59, respectively. The corresponding ratio for the creep recovery responses, which are characteristically smoother because the load cell is separated from any sources of noise associated with the loaded weight stack, is 0.94, 0.96, 0.96, and 1.04, respectively. The average value of *C*′/*C* for all of the creep responses analyzed in this study is 0.87, while the corresponding average value for the creep recovery responses is 0.94. Since the NTEP evaluation is generally based on the creep response, rather than the creep recovery response, use of the fitted curve to calculate the creep value would significantly reduce the effect of the random error.

The random error in the indicator readings depends on the characteristics of the indicating device, the dead-weight machine, and the load cell itself. Electrical noise, mechanical vibration, and weight movement all contribute to variations in the indicator readings. The instrumentation used at NIST to measure the voltage-ratio of the load cell strain gauge network contributes an uncertainty from random effects (expressed as a sample standard deviation) of about 0.0005 % of the reading at capacity load. In addition, some load cells exhibit more “noise” than others; inspection may reveal the “noise” to be dependent on the applied force. This effect may disappear if the same load cell is mounted in another deadweight machine capable of applying the same force. Clearly a complex load cell-deadweight machine interaction, possibly involving low level-driven harmonic oscillation, sometimes adds to the random error.

The random variation in the individual data points depends upon the indicating instrument sampling time that is chosen; for these creep measurements the sampling time is about 6 s. Increasing the sampling time may reduce the uncertainty due to random effects; however, this would involve the loss of some of the time variation information that the creep test is intended to measure. Such problems can be addressed through the use of curve-fitting calculations on readings taken continuously with relatively short sampling times.

### 5.3 Comparison of Creep Response and Creep Recovery Response

**Fig. 14:**
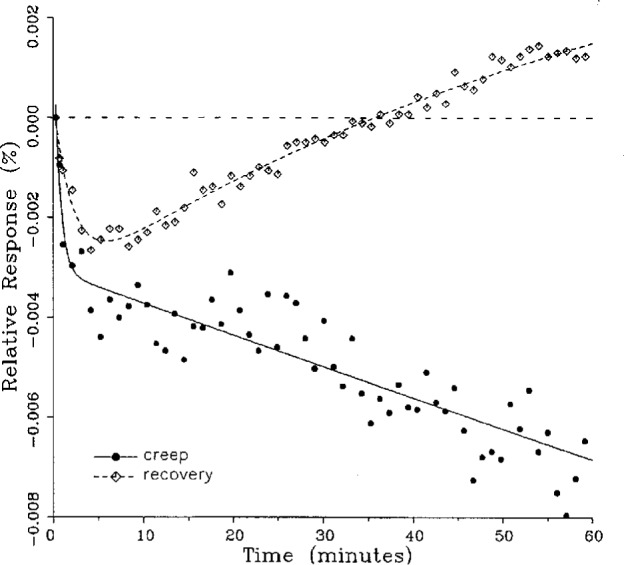
Creep response and creep recovery response of an S-shaped tension load cell of capacity 2.22 kN; temperature: −10.9 °C.

One purpose of this study is to determine whether the creep recovery response could be used in lieu of the creep response for design or evaluation purposes. Qualitative inspection of the creep and creep recovery pairs for all of the load cells that were tested indicates that, in 90 % of the cases, these two responses are similarly shaped; this judgement means that each term of Eq. (1) for the creep response has the same sign as the corresponding term for the creep recovery response, and that the transitions between the segments of the creep and creep recovery curves corresponding to these terms occur at about the same points in time. Figs. 3 to 12 show similarly shaped curves, for example, while in Fig. 13 the creep recovery does not have the same initial behavior as the creep response. An example of a more extreme case of dissimilar curves is shown in Fig. 14.

With one exception, the cases for which the curves are dissimilar are not correlated with any particular type of load cell construction, capacity, or test temperature. In addition, these cases generally involve only one of the three test temperatures for any one cell. The exception is one family of S-shaped tension load cells: for the five load cells tested in this family, one third of the creep-creep recovery pairs showed significant differences in shape.

Inspection of Figs. 3 to 14 indicates that, while the creep recovery response sometimes shows a greater magnitude of drift than the creep response, most often the reverse is true. If the creep values corresponding to the creep response and creep recovery response are calculated from the fitted curves according to Eq. (6), the ratio between the two values may be calculated for each pair of responses. For most cases, this ratio has a positive sign; for a few cases, as, for example, in Figs. 5 and 9a, the ratio is negative. Since the NTEP analysis is only concerned with the magnitude of the creep value, only the absolute values of the ratios are considered here. It is found that the recovery/creep ratio is less than 1.00 for 78 % of the all of the tests performed. The ratio falls between 0.60 and 1.00 for 59 % of the tests. The average of the absolute values of the recovery/creep ratios is 0.86, indicating that the creep recovery is, on the average, of a smaller magnitude than the creep value. Thus the creep response generally presents a more stringent test, when used for evaluation, than the creep recovery response.

In cases where the initial rate of creep is large, as is seen, for example, in Fig. 5, the difference between the two curves incorporates the uncertainty associated with the time of the 20 s reference reading, which appears as the first point in each of the figures. The creep test load is applied to the load cell by lowering the deadweight frame, which has already been loaded to a weight equal to the creep test load, onto the load cell by a hydraulic actuator or gear drive. The speed of the frame movement is adjusted to be slow enough to prevent shock to the load cell upon applying the load. The error in regulating the time between the load application or release and the sampling of the indicator for the reference reading may be as much as 2 s.

In the example of very rapid initial creep shown in Fig. 5, the contribution of the first exponential term in Eq. (1) is significant only for the first minute. An error of 2 s in locating the reference point here corresponds to a vertical displacement of about one-third of the distance between the minimum points of the two curves at the 1 min point. Thus the timing uncertainty cannot fully account for the difference between the creep response and creep recovery response shown in the figure. For most of the cases, which have smaller initial creep rates, the effect of the timing error is of less significance.

### 5.4 Comparison of NTEP and OIML Creep Evaluation

The NTEP procedure [1] specifies that the load cell creep be evaluated over a 60 min time period, while the OIML R 60 procedure [3] specifies a 30 min period. The OIML procedure requires two additional quantities to be determined: (1) the amount that the load cell creeps over a time period starting at 20 min and ending at 30 min after the initial reading; and (2) the minimum load output return, which is the difference between minimum load output readings before and after a maximum capacity load has been applied for 30 min.

The OIML R 60 procedure also differs from the NTEP procedure with respect to the time interval between the force application and the initial (reference) reading: the NTEP procedure specifies this time as 20 s, whereas the OIML procedure specifies a time interval that varies with the change in applied load. For the load cell capacities used in this study, this OIML specification varies from 15 s, for capacities of less than 980 N, to 50 s, for capacities greater than 98 kN. The tests conducted here used the 20 s interval specified by NTEP; however, the equation for each fitted curve can be used to estimate the load cell response at the reference time appropriate to either procedure.

Since the NIST NTEP tests involve a 60 min creep measurement followed by a 60 min creep recovery measurement, the OIML 30 min creep and 20 min to 30 min creep values can be computed from the NIST data; in addition, a minimum load output return (MLOR) at the end of 60 min of maximum load application can be computed. It is expected that, in general, the 60 min MLOR would be greater than or equal to the 30 min MLOR; thus an upper bound to the 30 min MLOR required by OIML can be computed from the NIST data. The 60 min, 30 min, and 20 min to 30 min creep values are determined from the fitted curves to Eq. (1).

These curves may be used to observe how the load cells tested here continued to creep after the first 30 min. To best compare this effect among the load cells without regard to the differing requirements for the initial time interval, let *R*_30/60_ denote the ratio of the 30 min creep to the 60 min creep, using a 20 s reference time for each case. The 30 min creep must then be less than or equal to the 60 min creep; it is found that the average value of *R*_30/60_ over all of the tests is 0.84. The ratio is equal to 1.00 for 15 % of the cases; *R*_30/60_ lies between 0.9 and 0.99 for 19 % of the cases, between 0.8 and 0.89 for 29 %, between 0.7 and 0.79 for 27 %, and less than 0.7 for 10 % of the cases. When *R*_30/60_ = 1.00, either the load cell response has reached a plateau by the 30 min point, as seen in Fig. 9b, or the maximum creep occurs in the early part of the creep response, as seen in Figs. 5, 7a, 8b, and 11. Lower ratios indicate more significant creep rates in the latter half of the response, as seen in Figs 6 and 9a, 9c, 14; the corresponding values of *R*_30/60_ for these figures are 0.56, 0.60, and 0.68, respectively.

There is a significant correlation between the OIML 20 min to 30 min creep value, which indicates how much the load creeps during this time period, and *R*_30/60_, which indicates how much of the cell creep occurs after the 30 min point. Letting *R*_20/30_ denote the ratio of the OIML 20 min to 30 min creep value to the OIML 30 min creep value, it is found, for instance, that for the cases where *R*_30/60_ lies between 0.9 and 0.99, *R*_20/30_ lies between 0.01 and 0.13 with an average value of 0.06; for the cases where *R*_30/60_ lies between 0.8 and 0.89, *R*_20/30_ lies between 0.04 and 0.25 with an average value of 0.12; and for the cases where *R*_30/60_ is less than 0.7, *R*_30/60_ lies between 0.20 and 0.66 with an average value of 0.32. Thus the OIML 20 min to 30 min creep value can, in general, identify those cases in which the OIML 30 min creep may fall significantly short of the NTEP 60 min creep.

The minimum load output return (MLOR), based on a 60 min application of maximum load, can be compared with the 60 min creep value by letting *R*_MLOR_ be the ratio of the MLOR value to the 60 min creep value. The average of the absolute values of *R*_MLOR_ is 1.05 for all of the tests conducted; however, only 43 % lie in the range of 0.9 to 1.1. These are the cases in which the creep response is monotonically increasing or decreasing with time. For example, in Figs. 3 and 4, *R*_MLOR_ has values of 0.99 and 1.03, respectively. In 37 % of the cases, *R*_MLOR_ is either less than 0.8 or greater than 1.2; in these cases the creep response is generally characterized by a large initial creep rate followed by a change in creep direction. For example, in Figs. 5 and 7a, *R*_MLOR_ has values of 2.1 and 0.10, respectively. Thus for almost 40 % of the time, the value of MLOR represents a different evaluation of the load cell creep behavior than is given by the maximum load cell creep.

The discussion of this section has been focused so far on how the physical parameters yielded by the NTEP and OIML creep analyses relate to each other. If the tolerances for these parameters that are currently permitted by the two programs are also considered, the actual outcomes of the NTEP and OIML evaluation procedures for the same set of load cells may be compared. This is accomplished here, making use of the fitted curves to compensate for the varying requirements for the initial reference time.

The NTEP and OIML creep tolerances depend on the load cell classification, which incorporates a specification of the maximum number of intervals, sometimes called scale divisions, into which the load cell measuring range may be divided. The number of divisions applicable for the load cells being discussed in this paper range from 3000 to 10 000. The creep tolerance is specified in terms of the load cell verification interval *v*, which is the value of one scale division. The NTEP creep tolerance on the 60 min creep is dependent on class and the number of divisions, and ranges from 1.5*v* to 5*v* for the load cells considered here. The OIML tolerances for class C, the OIML class which most closely compares to the NTEP classes, are considerable tighter: for the 30 min creep, the 20 min to 30 min creep, and the minimum load output return, these tolerances are 0.74*v*, 0.16*v*, and 0.53*v*, respectively.

Only 6 % of all of the load cells that were tested failed to meet the NTEP 60 min creep tolerance. In contrast, 62 % of the same load cells failed to meet the OIML 30 min creep tolerance, 41 % failed to meet the OIML 20 min to 30 min creep tolerance, and 79 % failed to meet the OIML tolerance for minimum load output return. All of the load cells that failed the 20 min to 30 min creep also failed the 30 min creep, and all of those that failed the 30 min creep also failed the minimum load output return. Under the current OIML tolerances, therefore, the minimum load output return presents the most severe test and the 20 min to 30 min creep presents the least severe, by about a factor of two. The NTEP 60 min creep evaluation is several times less severe than the OIML evaluations.

## 6. Conclusions

The multiple-term exponential equation given by Eq. (1) can be readily fitted to the creep data using curve-fitting routines in available data analysis software. Employing such a fitting procedure yields the creep response in a form that is most convenient for further analysis. Using the equation to calculate creep characteristics for evaluation testing minimizes the exaggeration of the creep value by noncreep related variation in the readings of the load cell output.

Shapes of creep and creep recovery curves are, in general, very similar. The magnitude of the creep recovery is, in general, less than the magnitude of the creep, with a difference, on the average, of more than 10 % of the creep value. For many families of load cells, the use of creep recovery data may be useful in production testing to monitor the creep performance, providing that actual creep tests representing a family do not indicate significantly dissimilar creep and creep recovery curves.

It is seen that load cells often have significant creep after the 30 min point following a load application; for example, in 37 % of the tests, the creep at 30 min is less than 0.8 of the creep at 60 min. Although the creep test of the OIML procedure does not yield creep response data beyond 30 min, the OIML placement of tolerance limits on the creep during the 20 min to 30 min period does provide a means of limiting the creep beyond 30 min.

The OIML measure of the minimum load output return (MLOR) after 30 min is not obtained by the NTEP creep test procedure. However, the MLOR after 60 min can be calculated from the NTEP data. This 60 min MLOR is seen to agree well with the 60 min creep for about half of the cases, in which the creep response is monotonically increasing or decreasing. The MLOR may significantly differ from the creep when the creep response has a large initial creep rate and a change in direction.

## Figures and Tables

**Fig. 7a:**
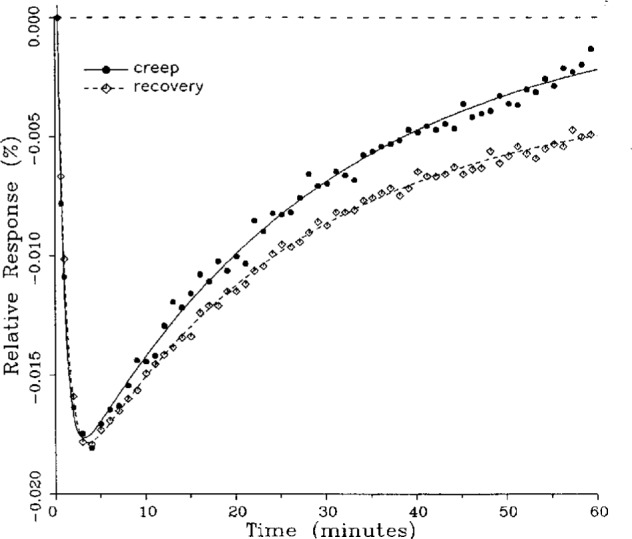
Creep response and creep recovery response of a shear-beam load cell of capacity 22.4 kN; temperature: 19.9 °C.

**Fig. 7b:**
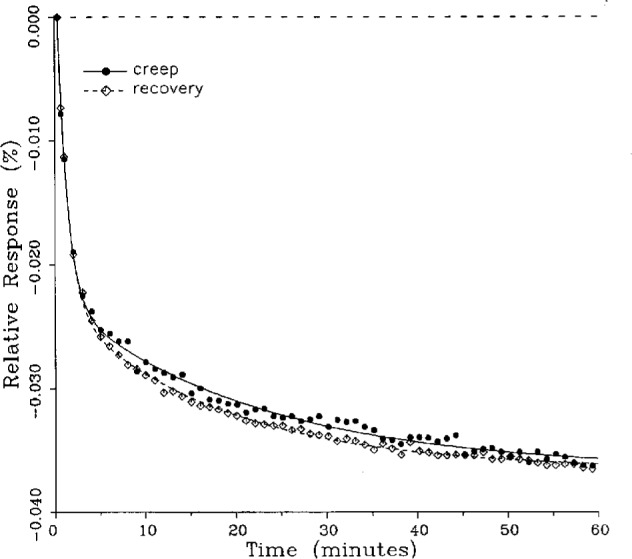
Same as for Fig. 7a but at a temperature of −9.6 °C.

**Fig. 7c:**
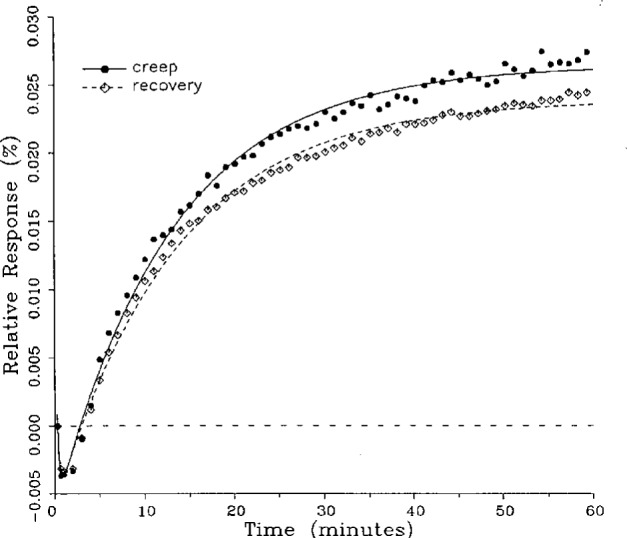
Same as for Fig. 7a but at a temperature of 39.6 °C.

**Fig. 8a:**
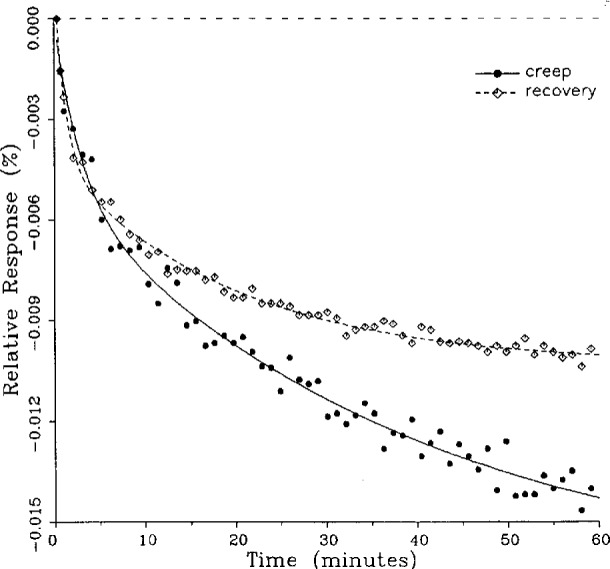
Creep response and creep recovery response of a shear-beam load cell of capacity 2.22 kN; temperature: 20.0 °C.

**Fig. 8b:**
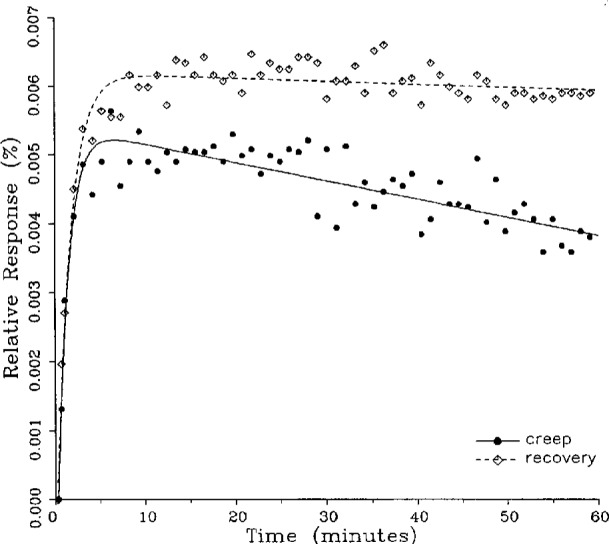
Same as for Fig. 8a but at a temperature of −9.9 °C.

**Fig. 8c:**
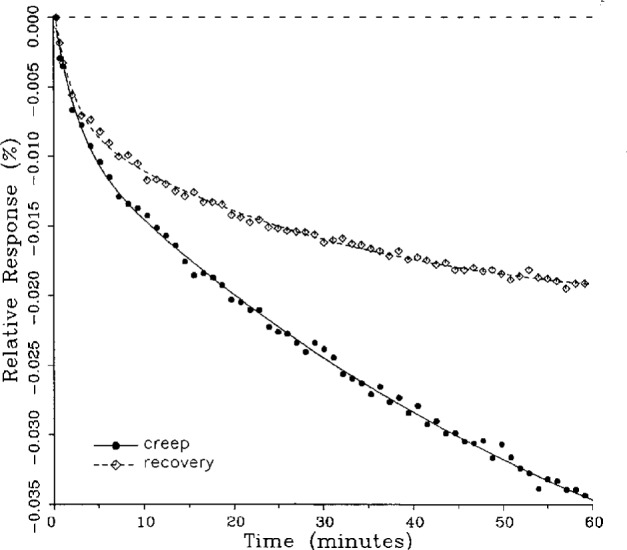
Same as for Fig. 8a but at a temperature of 40.0 °C.

**Fig. 9a:**
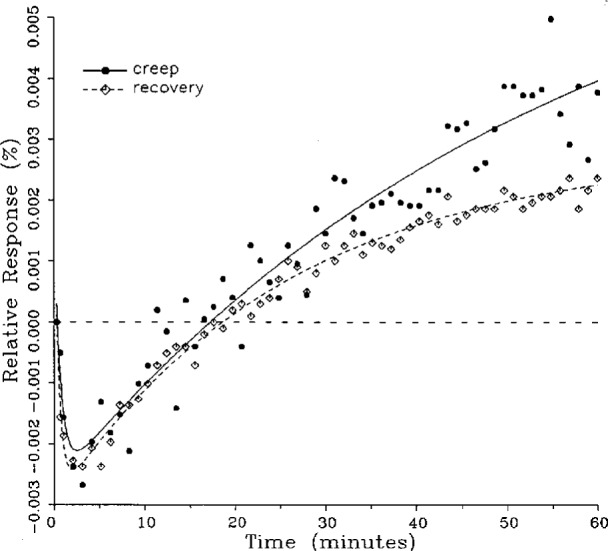
Creep response and creep recovery response of a single-point load cell of capacity 981 N; temperature: 20.2 °C.

**Fig. 9b:**
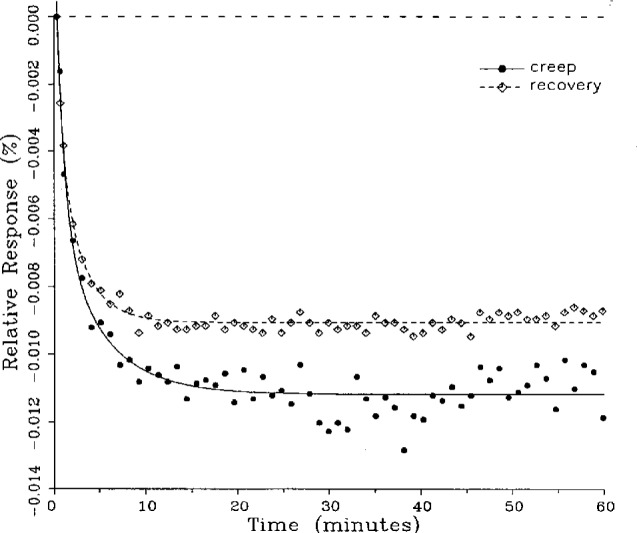
Same as for Fig. 9a but at a temperature of −8.1 °C.

**Fig. 9c:**
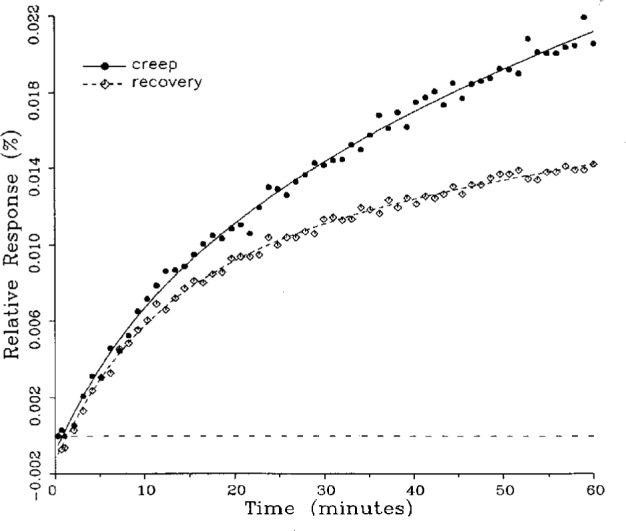
Same as for Fig. 9a but at a temperature of 39.1 °C.
